# Recording behaviour of indoor-housed farm animals automatically using machine vision technology: A systematic review

**DOI:** 10.1371/journal.pone.0226669

**Published:** 2019-12-23

**Authors:** Kaitlin Wurtz, Irene Camerlink, Richard B. D’Eath, Alberto Peña Fernández, Tomas Norton, Juan Steibel, Janice Siegford

**Affiliations:** 1 Department of Animal Science, Michigan State University, East Lansing, Michigan, United States of America; 2 Department of Farm Animals and Veterinary Public Health, Institute of Animal Welfare Science, University of Veterinary Medicine Vienna, Vienna, Austria; 3 Animal Behaviour & Welfare, Animal and Veterinary Sciences, Scotland’s Rural College (SRUC), Edinburgh, United Kingdom; 4 M3-BIORES– Measure, Model & Manage Bioresponses, KU Leuven, Leuven, Belgium; 5 Department of Fisheries and Wildlife, Michigan State University, East Lansing, Michigan, United States of America; National Veterinary School of Toulouse, FRANCE

## Abstract

Large-scale phenotyping of animal behaviour traits is time consuming and has led to increased demand for technologies that can automate these procedures. Automated tracking of animals has been successful in controlled laboratory settings, but recording from animals in large groups in highly variable farm settings presents challenges. The aim of this review is to provide a systematic overview of the advances that have occurred in automated, high throughput image detection of farm animal behavioural traits with welfare and production implications. Peer-reviewed publications written in English were reviewed systematically following Preferred Reporting Items for Systematic Reviews and Meta-Analyses (PRISMA) guidelines. After identification, screening, and assessment for eligibility, 108 publications met these specifications and were included for qualitative synthesis. Data collected from the papers included camera specifications, housing conditions, group size, algorithm details, procedures, and results. Most studies utilized standard digital colour video cameras for data collection, with increasing use of 3D cameras in papers published after 2013. Papers including pigs (across production stages) were the most common (n = 63). The most common behaviours recorded included activity level, area occupancy, aggression, gait scores, resource use, and posture. Our review revealed many overlaps in methods applied to analysing behaviour, and most studies started from scratch instead of building upon previous work. Training and validation sample sizes were generally small (mean±s.d. groups = 3.8±5.8) and in data collection and testing took place in relatively controlled environments. To advance our ability to automatically phenotype behaviour, future research should build upon existing knowledge and validate technology under commercial settings and publications should explicitly describe recording conditions in detail to allow studies to be reproduced.

## Introduction

Animal behaviour is implicitly used in an informal way every day by farmers to assess the health and welfare of the animals in their care [1]. More specifically, systematic and quantitative recordings of farm animal behaviour are made by researchers, veterinarians and farm assurance inspectors, for example using numerical scoring systems to record aspects of injury or lameness [2–4]. Various important behavioural traits have been shown to have sufficient heritability that genetic selection to modify them would be possible. Such traits include ease of handling, good maternal behaviour, and reduced harmful social behaviour or vices such as feather pecking in hens and tail biting in pigs [5,6]. To collect behavioural phenotypic information, breeders could carry out direct observation, behavioural testing, or make indirect / proxy measures of the consequences of behaviour such as skin lesion scores to indicate aggression [7] or tail damage scores to indicate that tail biting has occurred [8]. With a few exceptions, these have generally proved to be too time consuming and therefore too expensive to collect for widespread adoption by commercial livestock breeders.

In the examples described so far, recording animal behaviour is done manually via direct (or time-deferred by video) observations by human observers. In recent years, there has been a surge of interest in finding automated ways of recording behavioural traits. Devices which provide information about animal behaviour include: 1) Animal-based sensors fitted on (or sometimes in) the animal, such as tags, collars or bands containing devices which sense activity (tri-axial accelerometers; e.g., [9]) and/or location (Global Positioning System GPS, or radio-based proximity or triangulation) and/or contain information about animal identity (radio frequency identity, RFID) and 2) Environment-based sensors which can include RFID detectors, microphones, and a variety of camera technologies including monochromatic, colour, three-dimensional (3D), infra-red and thermal. In addition to animal behaviour, information streams useful in farm management include sensors which provide information about the farm environment or building control systems such as meteorological information, temperature, ventilation, the flow of water or feed, and the rate of production of eggs or milk. The concept of Precision Livestock Farming (PLF), includes the integration and interpretation of relevant sensor information enabling the management of individual animals through continuous real-time monitoring of health, behaviour, production, reproduction and environmental impact [10–12]. With the development of the Internet of Things (IoT, i.e. the interconnection between computing devices via the Internet), decision making can be better informed by connecting PLF information with other data streams, and components of farm management can be automated or even controlled remotely [13,14].

In this review we have chosen to focus on studies which attempt to automatically detect animal behaviour assess activity or identify individual animals using conventional (2D) monochromatic or colour cameras or 3D cameras [15]. We believe that cameras play an important role in the detection of animal behaviour data for PLF because: 1) a single unit can cover a group of animals and 2) machine vision analysis of camera data has the potential to reveal great behavioural detail and subtlety. Thermal cameras were excluded from the review because they provide detailed information on temperature and stress response but less about behaviour. Moreover, we found very few papers in which their use was automated (but see for example Franco et al., 2019 [16]).

**Fig 1:**
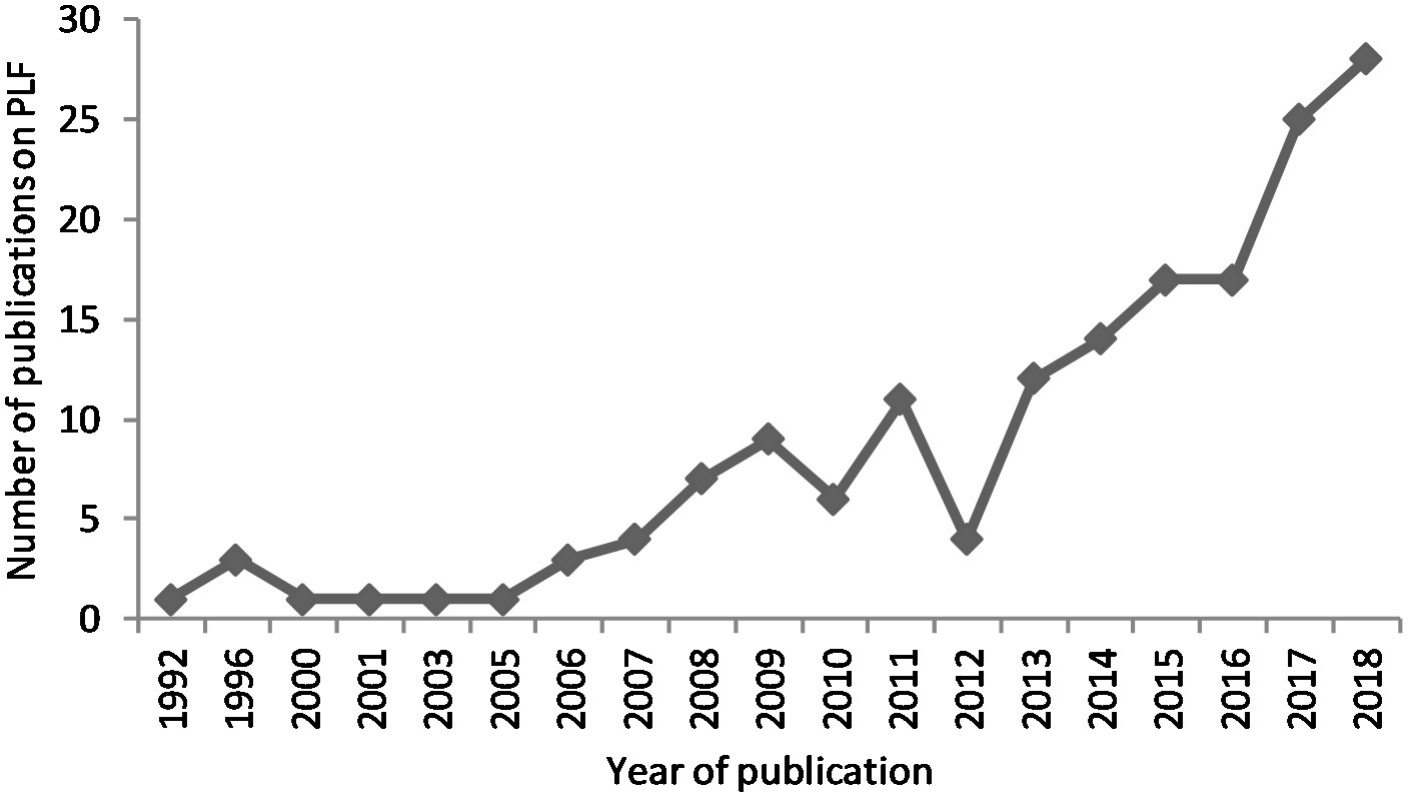
Historical chart, obtained via scopus, for the number of publications mentioning ‘Precision Livestock Farming’ in the title or abstract.

The use of cameras to automate the recording of behaviour has already been applied to species that are easy to manage in highly-controlled settings, for example movement tracking of colour-labelled laboratory rodents in small groups indoors under constant artificial light in standardised caging (e.g., Noldus, Ethovision [17]). Commercial farm conditions offer a number of challenges including group sizes and stocking density, unmarked individuals, variable lighting and background, and the possibility that the animal becomes soiled with dirt or faeces. Despite these challenges, rapid strides in the automatic recording of livestock and poultry behaviour have been made over the last few decades (Fig 1), and the rise of PLF as an interdisciplinary field combining technology with agriculture has resulted in studies published in a wide range of engineering and animal science journals. Studies published in engineering journals, or sometimes in national journals in native language specific to countries in for example Asia, South America, and Europe, are often not discovered by animal science researchers globally and may lack information that is important to allow others to replicate and build on the technology or to apply the technology practically in animal science settings. On the other hand, PLF studies published in animal science journals, sometimes claim novelty when it is not the case, and these claims may arise in part due to lack of knowledge of studies published outside their field. With an increasing number of researchers commencing studies using PLF there is a need for an overview of the current possibilities of technology to monitor animal behaviours that is accessible and comprehensible to readers across disciplines.

The aim of this review is therefore to provide a systematic review of the use of machine vision systems to automate the detection of animal behaviour traits in terrestrial farmed livestock and poultry using conventional and 3D cameras.

## Methodology

**Fig 2:**
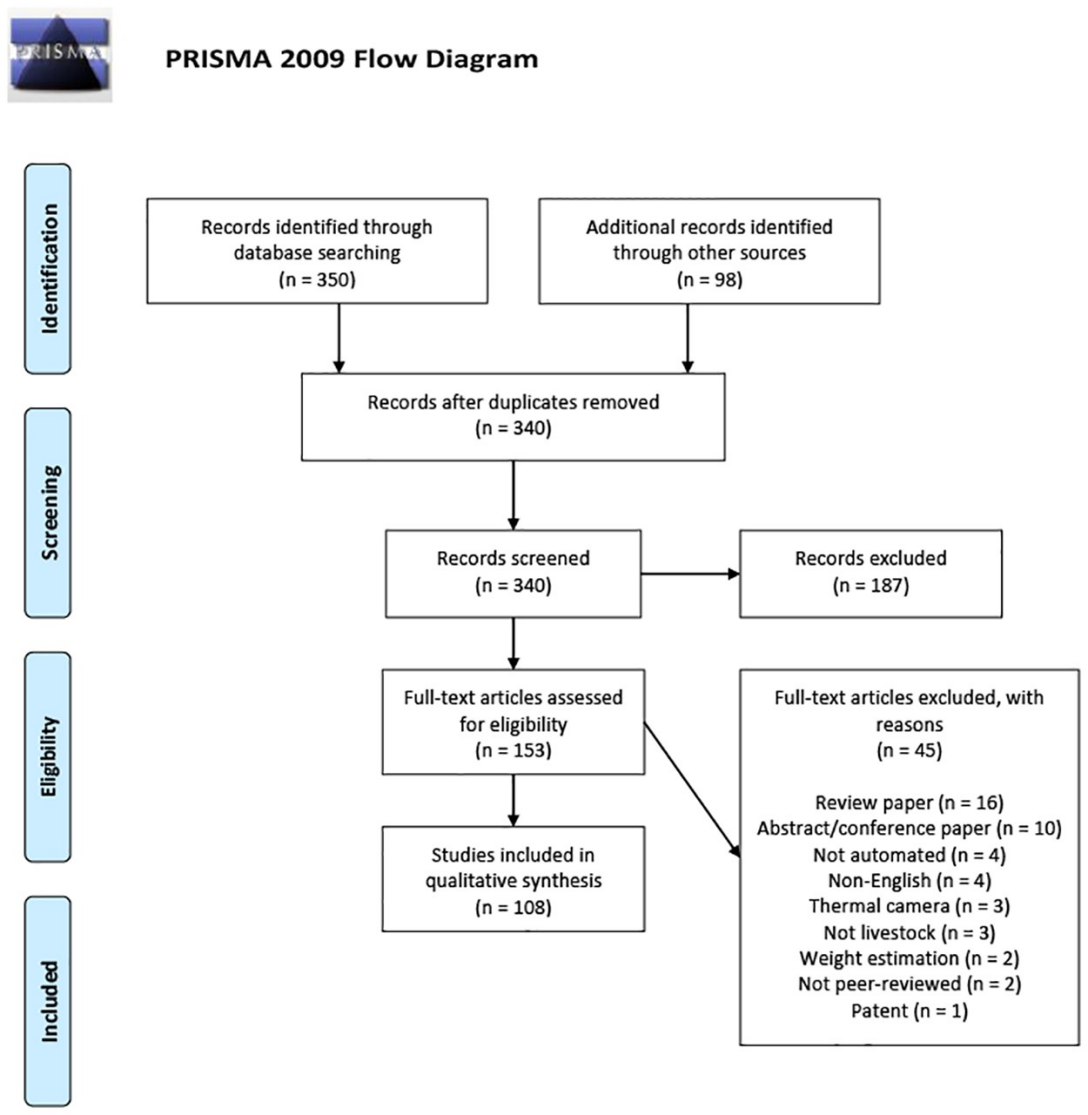
PRISMA flow diagram. *From*: Moher D, Liberati A, Tetzlaff J, Altman DG, The PRISMA Group (2009). *P*referred *R*eporting *I*tems for *S*ystematic Reviews and *M*eta-*A*nalyses: The PRISMA Statement, PLoS Med 6(7): e1000097. doi:10.1371/journal.pmed1000097. **For more information, visit** www.prisma-statement.org.

Literature was reviewed systematically by following Preferred Reporting Items for Systematic Reviews and Meta-Analyses (PRISMA) guidelines. PRISMA [18] specifies guidelines to be followed to collect an unbiased set of sources to use as the basis for the review question. The PRISMA steps for the current review are shown in Fig 2, along with the number of retrieved or retained publications at each stage.

### Eligibility criteria

Literature was evaluated regardless of publication year. Only peer-reviewed publications and peer-reviewed conference proceedings written in English were considered. Only studies on automated (machine vision) recording of livestock and poultry behaviour based on conventional or 3D cameras were included, thereby excluding any studies using animal-based (body-worn) sensors or on non-behavioural traits such as body weight.

### Information sources

The following databases were searched: Google Scholar, Web of Science, PubMed, AGRICOLA, AMiner, SciVerse, and ACM Digital Library. In addition, we searched the grey (non-commercially published) literature using Google to find additional sources of information. Literature from these databases was collected in March 2018.

### Search strategy and selection of publications

Each of the databases was searched for the following search term combinations: autom* + behavio* + livestock; autom* + behavio* + pig; autom* + behavio* + video + livestock; autom* + behavio* + video + pig; behavio* + detection + pig; autom* + behavio* + video detection + pig. An asterisk was used to automatically fill the search term to include related words such as behaviour, behavior, behavioural etc. If the database did not allow the use of the asterisk in the search term (which was the case for Google Scholar) then the term automated was used for autom*, and behavior or behaviour for behavio*.

Initially all livestock species were taken into account, by using the search term ‘livestock’. After it became apparent that pigs were the main study species for automated visual recording of behaviour, the more detailed search terms with ‘detection’ and ‘video detection’ were only used in combination with the word ‘pig’. Using the search combination ‘behavio* + autom* + video detection + pig’ in Google (not Google Scholar) gave 1,470,000 results, of which only the first 10 pages of results were examined in detail as additional pages of search results did not provide scientific papers meeting the search criteria.

All retrieved titles (n = 448) were recorded along with the database where they were found and search terms used to locate the article. All duplicates (n = 108) were removed based on author name, year and article title. Then, all titles and abstracts were screened. As the focus of the review was on visual sensing we removed all publications related to wearable monitors such as accelerometers (n = 187). From the remaining publications (n = 153), the full text was obtained and screened for eligibility using clear, non-arbitrary rules. After irrelevant publications (n = 45) were removed, the final publications were screened for relevant references not yet identified using the snowball method (i.e., searching references from relevant literature). Newly identified publications obtained through the snowball method (n = 41) were screened for the abstract and, if relevant, screened for the full text. The number of publications at each stage is given in the PRISMA flow diagram (Fig 2).

### Data extraction

From each full paper (n = 108), information was collected about the research methods and outcomes. The full description of data collection is given in the supplementary files (S1 Table). The data collected are presented in S2 Table. Briefly, information was recorded on the objective of the study, a detailed description of species and housing conditions, device type used for recording, the camera and lens specifications and settings, whether animals were tracked or not, whether tracked individuals were marked, type of behaviour recorded, the data processing method, the method used to validate automatic detection, and results such as accuracy and precision.

## Results

The results of our systematic search of the literature for studies using visual sensing yielded papers covering only pigs, poultry and dairy cattle. Precision livestock farming publications on other livestock species were sensor-based, and thus were excluded from this review. We first review the main devices and techniques used in general. Then, for each species, we describe the methods used and types of behaviour recorded by specific age category, if relevant.

### Devices

Studies up to 2014 mainly use 2D (i.e., digital) cameras, monochrome or colour, for recordings. Since 2014 the use of 3D (i.e., depth) cameras has increased (S1 Table). Traditional animal behaviour video recordings (i.e., those decoded by human observers) are often made by cameras placed on a rail above the pen, creating an angular view. This creates difficulties when automating analysis of recordings [19], and nowadays almost all studies collecting video for automated decoding place cameras directly above the pen to create a top-down view (79 out of 108 studies report top-down-view, S1 Table).

#### Digital cameras (2D)

Digital 2D video cameras are in widespread use, readily available and relatively cheap. Colour 2D digital cameras essentially work via a light-sensing chip known as a CCD (charge-coupled device) that detects the brightness of three filtered colour channels (red, green and blue, abbreviated to RGB) at every pixel. Several compressed file formats (such as MPEG-4) are available to efficiently store the image data they generate. For some applications, simpler monochrome video cameras, which use a CCD to only record light intensity at each pixel are sufficient. Monochrome cameras typically have greater light sensitivity and are thus more ideal for recording under lower light conditions than colour cameras. 2D cameras, however, are limited in application as they provide only a flat projection of the animal [20] and are also influenced by distance, specific wavelength, and any applied filters [21].

#### Depth cameras (3D)

3D cameras allow for broader application as they can capture additional information, depth, which represents the vertical distance between target and camera, and are less prone to environmental influences. Our review identified 13 papers using 3D cameras: 10 on pigs, two on dairy cattle and one on poultry. 3D cameras are more expensive and typically provide lower image resolution (i.e., fewer pixels per area), but they have a number of advantages over conventional 2D colour video cameras. They can operate regardless of the visual light environment, including in total darkness, and they are unaffected by changing light conditions including changes in contrast and shadow, and are less prone to errors due to occlusion [22]. On farm, variable light conditions frequently occur over the course of a day as part of normal light and dark schedules or due changing amounts of sunlight entering buildings via windows or open sides, and part or all of the surface of an animal can become soiled with dirt, mud or faeces, changing its colour and relative contrast against the background (which itself may also become soiled). Both variable lighting and changes due to soiling can create challenges for successful machine vision segmentation based on differences in colour [23].

3D cameras make segmentation (finding the animal against the background, described in more detail below) much easier because depth information means that anything that is closer to the camera than the floor or pen fixtures must be an animal. In commercial farm buildings, dust in the air (and insects such as flies) can present a challenge to even this seemingly basic first step of segmentation [24]. Once animals have been identified against the environmental background, posture detection is also simpler with a 3D camera, since a lying animal is further away from an overhead camera than a standing animal [25]. 3D technology also opens the possibility to reconstruct the geometry of animals’ bodies and link abnormal morphological changes to behavioural changes [26–28]. Most 3D studies have used the relatively cheap Microsoft Kinect 3D camera system, ideal for research use since it also came with a package of software for developers. However, the Kinect was discontinued in late 2017, and now researchers are turning to other similar cheap and user-friendly systems such as the Intel RealSense. Industrial-specification 3D cameras are also available, which are more robust to the dust, ammonia and high pressure water cleaning systems on commercial pig farms, and these were used by D’Eath et al. in a study describing the use of a 3D camera system to detect tail posture as an early-warning sign of tail biting [8].

### Techniques for extracting data

The challenge of detecting the behaviour of animals can broadly be divided into several steps: segmentation, feature extraction, and behaviour definition. Segmentation is essentially separating the animal from a non-animal background. Segmentation can also include the detection and tracking of features specific to certain individual animals, such as segmenting pixels based upon colour, intensity, texture, and or location. Feature extraction encompasses separation of individual animals from each other, orientation of those animals, and aspects of detecting motion and tracking animals across frames over time. Behavioural definition is the identification and quantification of specific activity patterns, postures, gaits or behaviours shown by the animals of interest. Algorithms that have been used for these tasks across the reviewed papers are listed in Table 1, classified by the objective for which they have been used. As many of these papers describe work at fairly early research stages with respect to automatic data extraction, we make no distinction between systems that achieve real-time ‘live’ data processing and those that collect sample data sets for later ‘off-line’ processing and analysis.

**Table 1:** Algorithms described in the reviewed literature and objectives for which they were used.

**Segmentation**		
**Objective**	**Method**	**References**
Aggregated points belonging to the same object	Labelling connected domain	Chen et al. 2017
Background subtraction	Channel features	Nilsson et al. 2014, 2015a,b
Background subtraction	Isomap algorithm	Zhu et al. 2014
Background subtraction	Mode-base	Tsai et al. 2014, 2015
Background subtraction	Non-linear colour combination	Poursaberi et al. 2010; Van Hertem et al. 2014^a^
Background subtraction	Reference image	Ahrendt et al., 2011; Kim et al. 2017a^a^; Lindt et al. 2015; Nasirahmadi et al. 2015, 2016, 2017b,c; Oczak et al. 2014; Hansen et al. 2018^a^; Pluk et al. 2012; Song et al. 2008; Souza et al. 2009; Aydin et al. 2013; Leroy et al. 2006
Background subtraction	Gaussian mixture model	Ahrendt et al., 2011; Baek et al. 2017; Chung et al. 2014
Background subtraction	Maximum entropy	Chen et al. 2017; Zhu et al. 2009, 2017
Background subtraction	Otsu's	Chung et al. 2014; Costa et al. 2014, 2015; Kashiha et al. 2013a,b,c, 2014; Kim et al. 2017a,b,c^a^; Kongsro et al. 2013; Lind et al. 2015; Ma et al. 2016; Nasirahmadi et al. 2015, 2016, 2017b,c; Ott et al. 2014; Shao et al. 2008; Shao, Xin & Harmon, 1997; Xin et al. 2002; Yu et al. 2015; Zhu et al. 2015; Cangar et al. 2007, 2008; Sergeant et al. 1998; Zhuang et al. 2018
Background subtraction	Frame difference	Chung et al. 2014; Van Hertem et al. 2014^a^; Aydin et al. 2010
Connect separate regions	Small neighborhoods	Khoramshahi et al. 2014
Contrast enhancement	CLAHE	Zheng et al. 2018^a^
Correct for lens distortion	Polynomial models	Gronskyte et al. 2015; Lind et al. 2015
Define objects	Blob	Fernández-Carrión et al. 2017; Gronskyte et al. 2013, 2015, 2016
Discriminate between regions	Region-based	Ju et al. 2018a
Discriminate different region between frames	Outline-based	Ju et al. 2018a
Discriminate objects	Hysteresis discrimination	Sergeant et al. 1998; Zhuang et al. 2018
Enhance spatial correlation and colour similarities	Mahalanobis distance	Ahrendt et al., 2011
Estimation of objects and movement	Optical flow	Fernández-Carrión et al. 2017; Gronskyte et al. 2013, 2015, 2016; Colles et al. 2015; Dawkins et al. 2009, 2012
Group pixels	Line-coincidence	Perner, 2001
Image enhancement	Histogram equalization	Chen et al. 2017; Kashiha et al. 2013a,b,c; Lao et al. 2016^a^; Ott et al. 2014; Weixing et al. 2010b; Zhu et al. 2017
Reduce noise	Median filter	Lind et al. 2015; Zheng et al. 2018^a^; Nakarmi et al. 2014
Reduce number of pixels	Spatio-temporal interpolation	Kim et al. 2017a,b^a^
Remove small objects and soften of edges	Morphological filtering	All
Segmentation of thermal images	Topographic surface	Kim et al, 2017c
Separate objects from background	Colour decorrelation	Gronskyte et al. 2015; Aydin et al. 2013; Pereira et al. 2013
Separate touching objects	Watershed	Kim et al, 2017c; Oczak et al. 2016; Nakarmi et al. 2014
Separate touching objects	K-means	Zhuang et al. 2018
**Feature extraction**		
**Objective**	**Method**	**References**
2D boundary tracing	Moore neighborhood	Kashiha et al. 2013b,c
Activity estimation	Intensity difference	Costa et al. 2013, 2014; Oczak et al. 2014; Ott et al. 2014; Sergeant et al. 1998
Contour description	Fourier coefficients	Kashiha et al. 2013b,c; Shao, Xin & Harmon, 1997; Weixing et al. 2010a
Contour description	CowEdge	Van Hertem et al. 2014
Define boundaries between two regions	Hough transform	Baek et al. 2017; Zhuang et al. 2018
Define center point of an area	Medial axis transform	Ju et al. 2017a
Descriptor for local structure in gray level	Local binary pattern	Huang et al. 2018
Determine convex points in a curve	Convex hull algorithm	Ju et al. 2017b
Enhanced shapes	Anisotropic diffusion filter	Nakarmi et al. 2014
Extract contours	Wavelet edge detection	Ma et al. 2016
Extract contours	Zernike moments	Zhu et al. 2015
Extract edge information	Canny operator	Baek et al. 2017; Kim et al. 2017b^a^; Zhu et al. 2015
Extract edge information	Laplace operator	McFarlane and Schofield 1995
Extract features from object	Area fitting	Chen et al. 2017
Extract features from optical flow vectors	Doane’s formula	Chen et al. 2017
Extract features from optical flow vectors	Modified angular histograms	Fernández-Carrión et al. 2017; Gronskyte et al. 2013, 2015, 2016
Extraction of texture features	Gabor filters	Huang et al. 2018
Feature extraction	Active shape algorithm	Hansen et al. 2018^a^; Pluk et al. 2012; Song et al. 2008; Souza et al. 2010;Cangar et al. 2007, 2008; Poursaberi et al. 2010
Group objects	Delaunay triangulation	Nasirahmadi et al. 2015, 2016, 2017b
Link objects with regions	Kuhn-Munkres algorithm	Yu et al. 2015
Locate object	Ellipse fitting	Kashiha et al. 2013a,b,c, 2014; Lind et al. 2015; Ma et al. 2016; McFarlane and Schofield 1995; Nasirahmadi et al. 2015, 2016, 2017b,c; Oczak et al. 2016; Zhuang et al. 2018
Map arrays of features	Elastic net regularized logistic regression	Nilsson et al. 2014, 2015a,b
Minimal distance between points in a region of interest	Euclidean distance	Chen et al. 2017; Nasirahmadi et al. 2015, 2016, 2017b,c; Shao et al. 2008; Zhu et al. 2017; Leroy et al. 2005
Motion detection	Shading model	Shao et al. 2008
Motion detection	Motion history image	Viazzi et al. 2014; Ahn et al. 2018
Motion detection	XOR operation	Xin et al. 2002
Motion smoothing	Moving average filter	Fernández-Carrión et al. 2017; Gronskyte et al. 2013, 2015, 2016; Lao et al. 2016^a^
Multi-object contour extraction	Morphology processing	Ma et al. 2016
Outliers filtering	Dynamic linear model	Kristensen & Cornou, 2011
Outline shapes	Point distribution model	Leroy et al. 2005, 2006
Region definition	Back posture measurement	Van Hertem et al. 2015
Select periods of interest in set of images	Key frames	Wang et al. 2015; Chen et al. 2017
Separation of adjacent regions	Concave-convex points	Baek et al. 2017; Kim et al, 2017c; Weixing et al. 2010b; Zhuang et al. 2018
Separation touching objects	Normal surfaces in 3D	Matthews et al. 2017^a^
Track object between frames	Hungarian method	Matthews et al. 2017^a^
Track objects	Support maps	Ahrendt et al., 2011
Track objects	EthoVision software	Kulikov et al. 2014^a^; Suster et al. 2001; Fraess et al. 2016
Track objects	Particle filter	Fujii et al. 2008
Track objects movement	Linear angular motion	Kashiha et al. 2013c
**Behavioural definition**		
**Objective**	**Method**	**References**
Align time-series of features	Dynamic time wrapping	Hunag et al. 2018
Classification	Support vector machine	Hunag et al. 2018; Lee et al. 2016; Weixing et al. 2010a; Zhu et al. 2014, 2015; Zhuang et al. 2018
Classification	Neural networks	Khoramshahi et al. 2014; Oczak et al. 2014; Shao, Xin & Harmon, 1997; Zheng et al. 2018
Classification	Linear discriminant Analysis	Viazzi et al. 2014
Classification	Viola-Jones algorithm	Porto et al. 2013, 2015
Classification	Weka software	Pereira et al. 2013
Cluster together regions with similar properties	Hierarchical clustering	Chen et al. 2017
Establish relation between variables	Transfer functions	Kashiha et al. 2013a; Oczak et al. 2016; Leroy et al. 2005, 2006; Youssef et al. 2015
Reduce dimensionality	Principal component analysis	Hunag et al. 2018; Kongsro et al. 2013
**Segmentation**		
**Method**	**Objective**	**References**
Labelling connected domain	Aggregated points belonging to the same object	Chen et al. 2017
Channel features	Background subtraction	Nilsson et al. 2014, 2015a,b
Isomap algorithm	Background subtraction	Zhu et al. 2014
Mode-base	Background subtraction	Tsai et al. 2014, 2015
Non-linear colour combination	Background subtraction	Poursaberi et al. 2010; Van Hertem et al. 2014
Reference image	Background subtraction	Ahrendt et al., 2011; Kim et al. 2017a; Lindt et al. 2015; Nasirahmadi et al. 2015, 2016, 2017b,c; Oczak et al. 2014; Hansen et al. 2018; Pluk et al. 2012; Song et al. 2008; Souza et al. 2009; Aydin et al. 2013; Leroy et al. 2006
Gaussian mixture model	Background subtraction	Ahrendt et al., 2011; Baek et al. 2017; Chung et al. 2014
Maximum entropy	Background subtraction	Chen et al. 2017; Zhu et al. 2009, 2017
Otsu's	Background subtraction	Chung et al. 2014; Costa et al. 2014, 2015; Kashiha et al. 2013a,b,c, 2014; Kim et al. 2017a,b,c; Kongsro et al. 2013; Lind et al. 2015; Ma et al. 2016; Nasirahmadi et al. 2015, 2016, 2017b,c; Ott et al. 2014; Shao et al. 2008; Shao, Xin & Harmon, 1997; Xin et al. 2002; Yu et al. 2015; Zhu et al. 2015; Cangar et al. 2007, 2008; Sergeant et al. 1998; Zhuang et al. 2018
Frame difference	Background subtraction	Chung et al. 2014; Van Hertem et al. 2014; Aydin et al. 2010
Small neighborhoods	Connect separate regions	Khoramshahi et al. 2014
CLAHE	Contrast enhancement	Zheng et al. 2018
Polynomial models	Correct for lens distortion	Gronskyte et al. 2015; Lind et al. 2015
Blob	Define objects	Fernández-Carrión et al. 2017; Gronskyte et al. 2013, 2015, 2016
Region-based	Discriminate between regions	Ju et al. 2018a
Outline-based	Discriminate different region between frames	Ju et al. 2018a
Hysteresis discrimination	Discriminate objects	Sergeant et al. 1998; Zhuang et al. 2018
Mahalanobis distance	Enhance spatial correlation and colour similarities	Ahrendt et al., 2011
Optical flow	Estimation of objects and movement	Fernández-Carrión et al. 2017; Gronskyte et al. 2013, 2015, 2016; Colles et al. 2015; Dawkins et al. 2009, 2012
Line-coincidence	Group pixels	Perner, 2001
Histogram equalization	Image enhancement	Chen et al. 2017; Kashiha et al. 2013a,b,c; Lao et al. 2016; Ott et al. 2014; Weixing et al. 2010b; Zhu et al. 2017
Median filter	Reduce noise	Lind et al. 2015; Zheng et al. 2018; Nakarmi et al. 2014
Spatio-temporal interpolation	Reduce number of pixels	Kim et al. 2017a,b
Morphological filtering	Remove small objects and soften of edges	All
Topographic surface	Segmentation of thermal images	Kim et al, 2017c
Colour decorrelation	Separate objects from background	Gronskyte et al. 2015; Aydin et al. 2013; Pereira et al. 2013
Watershed	Separate touching objects	Kim et al, 2017c; Oczak et al. 2016; Nakarmi et al. 2014
K-means	Separate touching objects	Zhuang et al. 2018
**Feature extraction**		
**Method**	**Objective**	**References**
Moore neighborhood	2D boundary tracing	Kashiha et al. 2013b,c
Intensity difference	Activity estimation	Costa et al. 2013, 2014; Oczak et al. 2014; Ott et al. 2014; Sergeant et al. 1998
Fourier coefficients	Contour description	Kashiha et al. 2013b,c; Shao, Xin & Harmon, 1997; Weixing et al. 2010a
CowEdge	Contour description	Van Hertem et al. 2014
Hough transform	Define boundaries between two regions	Baek et al. 2017; Zhuang et al. 2018
Medial axis transform	Define center point of an area	Ju et al. 2017a
Local binary pattern	Descriptor for local structure in gray level	Huang et al. 2018
Convex hull algorithm	Determine convex points in a curve	Ju et al. 2017b
Anisotropic diffusion filter	Enhanced shapes	Nakarmi et al. 2014
Wavelet edge detection	Extract contours	Ma et al. 2016
Zernike moments	Extract contours	Zhu et al. 2015
Canny operator	Extract edge information	Baek et al. 2017; Kim et al. 2017b; Zhu et al. 2015
Laplace operator	Extract edge information	McFarlane and Schofield 1995
Area fitting	Extract features from object	Chen et al. 2017
Doane’s formula	Extract features from optical flow vectors	Chen et al. 2017
Modified angular histograms	Extract features from optical flow vectors	Fernández-Carrión et al. 2017; Gronskyte et al. 2013, 2015, 2016
Gabor filters	Extraction of texture features	Huang et al. 2018
Active shape algorithm	Feature extraction	Hansen et al. 2018; Pluk et al. 2012; Song et al. 2008; Souza et al. 2010;Cangar et al. 2007, 2008; Poursaberi et al. 2010
Delaunay triangulation	Group objects	Nasirahmadi et al. 2015, 2016, 2017b
Kuhn-Munkres algorithm	Link objects with regions	Yu et al. 2015
Ellipse fitting	Locate object	Kashiha et al. 2013a,b,c, 2014; Lind et al. 2015; Ma et al. 2016; McFarlane and Schofield 1995; Nasirahmadi et al. 2015, 2016, 2017b,c; Oczak et al. 2016; Zhuang et al. 2018
Elastic net regularized logistic regression	Map arrays of features	Nilsson et al. 2014, 2015a,b
Euclidean distance	Minimal distance between points in a region of interest	Chen et al. 2017; Nasirahmadi et al. 2015, 2016, 2017b,c; Shao et al. 2008; Zhu et al. 2017; Leroy et al. 2005
Shading model	Motion detection	Shao et al. 2008
Motion history image	Motion detection	Viazzi et al. 2014; Ahn et al. 2018
XOR operation	Motion detection	Xin et al. 2002
Moving average filter	Motion smoothing	Fernández-Carrión et al. 2017; Gronskyte et al. 2013, 2015, 2016; Lao et al. 2016
Morphology processing	Multi-object contour extraction	Ma et al. 2016
Dynamic linear model	Outliers filtering	Kristensen & Cornou, 2011
Point distribution model	Outline shapes	Leroy et al. 2005, 2006
Back posture measurement	Region definition	Van Hertem et al. 2015
Key frames	Select periods of interest in set of images	Wang et al. 2015; Chen et al. 2017
Concave-convex points	Separation of adjacent regions	Baek et al. 2017; Kim et al, 2017c; Weixing et al. 2010b; Zhuang et al. 2018
Normal surfaces in 3D	Separation touching objects	Matthews et al. 2017
Hungarian method	Track object between frames	Matthews et al. 2017
Support maps	Track objects	Ahrendt et al., 2011
EthoVision software	Track objects	Kulikov et al. 2014; Suster et al. 2001; Fraess et al. 2016
Particle filter	Track objects	Fujii et al. 2008
Linear angular motion	Track objects movement	Kashiha et al. 2013c
**Behavioural definition**		
**Method**	**Objective**	**References**
Dynamic time wrapping	Align time-series of features	Hunag et al. 2018
Support vector machine	Classification	Hunag et al. 2018; Lee et al. 2017; Weixing et al. 2010a; Zhu et al. 2014, 2015; Zhuang et al. 2018
Neural networks	Classification	Khoramshahi et al. 2014; Oczak et al. 2014; Shao, Xin & Harmon, 1997; Zheng et al. 2018
Linear discriminant Analysis	Classification	Viazzi et al. 2014
Viola-Jones algorithm	Classification	Porto et al. 2013, 2015
Weka software	Classification	Pereira et al. 2013
Hierarchical clustering	Cluster together regions with similar properties	Chen et al. 2017
Transfer functions	Establish relation between variables	Kashiha et al. 2013a; Oczak et al. 2016; Leroy et al. 2005, 2006; Youssef et al. 2015
Principal component analysis	Reduce dimensionality	Hunag et al. 2018; Kongsro et al. 2013

^a^Denotes publications utilizing 3D cameras.

Useful metrics of behaviour can be obtained from each of these steps. Basic measures of how and when space is being occupied, and thus certain types of resource use can be inferred simply from segmentation (e.g., feeding based on presence of a pig near the feeder), although more accuracy is possible if some feature extraction is added (e.g., determining that a moving ‘blob’ is a single standing/walking pig, tracking that it has moved towards a feeder and entered it face first would give us more confidence that we have genuinely recorded feeder use). Tracking the motion of animals over frames, even if imperfectly, can generate further behavioural metrics such as activity and acceleration. Activity measures can form the basis of more accurate detection of specific behaviours such as walking, running, play or aggressive behaviours or problems such as lameness. Various studies in our review have reached different points along this progression. Only rarely, however, do the authors explicitly describe how their method represents a refinement or advance over a previous approach to the same problem.

In the following three sections, we review the literature for automated detection of behaviour using machine vision technology in 1) pigs, 2) poultry and 3) dairy cattle in turn. Each section begins with a brief overview that classifies the studies we found, followed by a more detailed discussion, grouping together studies that followed a similar underlying approach.

### Use of automated behaviour detection in pigs

The majority of studies attempting to use machine vision technology to automatically detect behaviour in pigs have been conducted on groups of growing pigs, with a few studies targeting singly-housed sows or suckling piglets. Conventional 2D cameras were the most common camera type used (n = 54), with more recent studies beginning to use 3D depth cameras (n = 10). A top-down view was the most common camera position (55 studies), while five studies used an angled top-down view, three studies viewed animals from the side, and one study did not mention camera location. All monitoring took place in indoor environments. Eighteen studies were conducted on commercial farms, 23 in indoor research facilities, 21 were conducted in specially equipped test pens, and two studies observed pigs in a slaughterhouse. Floor materials, and thus colour of the background, varied greatly among studies. Most studies had concrete flooring (n = 35) that was ranged from solid to partially slatted or fully slatted. Nine studies had slatted floors of various colours, while six studies had floors bedded with a substrate. Fourteen studies failed to provide any details related to floor type or colour, which was surprising given how central background colour is to the functioning of machine vision algorithms that must subtract this element. Of the few studies that made any mention of lighting conditions, descriptions were typically vague such as ‘varied’, ‘sunny’, or ‘natural’ rather than specifying light intensity or source type. Group sizes used for testing were generally small (mean = 10.3 individuals, median = 9) and ranged from one to 40 pigs in a pen. Total study population size was larger (mean = 43.4, median = 17, range = 1–667). Information generated by the tracking software varied from the basics of segmentation of individuals (11 studies), to tracking activity or resource use (29 studies), to detecting postures or gait scores (10 studies) and moving up to more advanced detection of specific behaviours (11 studies).

#### Image segmentation

As described previously, segmentation, or the process of distinguishing individual animals from their background, is a necessary initial step in machine vision methodology. The first attempt to use segmentation in the automatic detection of pigs from images was in the nineties when McFarland and Schofield attempted to track recently weaned pigs [29]. They distinguished piglets from their background by modelling them as ellipses based on blob edges obtained from image differencing between successive frames; however, they encountered difficulties because the frame speed of the camera was too low to accurately capture the piglets’ rapid movements. This issue has now been overcome with improvements to cameras, and high-speed cameras can now capture hundreds of frames per second.

Additional early segmentation studies applied the technique to assess thermal comfort of piglets [30–32]. Automated monitoring of thermal comfort has potential to improve animal welfare by recognizing pigs’ behavioural attempts to thermoregulate and responding with appropriate environmental adaptations. The initial studies examining this problem first created binarized images using thresholding, edge detection, and morphological filtering techniques [33]. Extraction of features using a combination of Fourier coefficients, moments, perimeter and area were able to successfully identify spacing between the pigs—a behavioural indicator of whether pigs were cold (tightly clustered together) or too warm (widely spaced apart). About a decade later, in another effort to measure thermal comfort, Shao & Xin developed a method to identify individual pigs lying down using a predefined threshold of intensity ratio to determine if motion had occurred between successive frames [34]. Any moving pigs were removed, along with small objects, and then feature extraction was performed on the resulting images to measure the compactness of the lying pigs.

Segmentation to resolve touching pigs into separate individuals can be difficult, as classical segmentation techniques cannot recognize boundaries between pigs. One research group proposed a method using concave points and edge information generated from continuous video frames transformed into a one-dimensional time-series [35–37]. This allowed for accurate separation of touching pigs in a crowded room environment. An alternative method was proposed by Oczak et al. [38]. This group employed a Watershed algorithm [39] to binarized greyscale images to find boundaries between piglets and between piglets and a sow (irrespective of their shape). This allowed researchers to accurately estimate the number of piglets at farrowing.

Otsu’s commonly used greyscale method, developed in the 1970s, works well in controlled environments, with large contrast between the pigs and the background [40]. However, this method struggles in real world applications with variable lighting, multi-coloured pigs, or non-contrasting pig and floor colours. Nilsson et al. reported an improved method for segmentation of pigs [41] that processed the raw colour image in multiple different ways by looking for colour contrast, areas of maximum colour change and colour gradients in various directions, as well as a max-min filter and a modification of Otsu’s method. The optimal combination of these channels was then found using a learning-based approach to structured prediction and fitting an elastic net regularized logistic regression. Nilsson et al. validated their approach by estimating the number of pigs in a region of the pen (the dunging area) in a 5-minute video sequence and found good agreement when comparing this against human observer counts [42,43].

Another advanced segmentation technique was developed by Ma et al. to better cope with the light changes that are common in pig barns [44]. Their method used adaptive elliptic block and wavelet edge detection after performing two-dimensional Otsu threshold segmentation. To investigate pig boundary detection in dirty pen environments with insufficient lighting, Buayai et al. applied adaptive thresholding using an integral image for segmentation and used adaptive partitioning with connected components [45]. This step was followed by using the maximum entropy threshold of each partition, and merging the results from both steps, creating a more robust segmentation procedure. A further segmentation study implemented a neural network classifier (supervised machine learning technique) to identify individuals within a group [46]. This method demonstrated effective segmentation of a sow’s body, even when she was partially occluded by the crate elements. Tu et al. addressed the issues of dynamic background objects, light changes, and motionless foreground objects in their study of sows in free-farrowing pens using a Gaussian mixture model for background subtraction and dyadic wavelength transformation, and then performed tracking using the centre of mass [47].

#### Occupation and movement

Individual and group activity levels and location within a pen can be estimated using pixel analysis obtained from digital video. An occupation index can be obtained by measuring the percentage of pixels above a defined threshold. This can be done at the pen level, or on focal regions within the pen. An activity index can also be obtained by measuring the amount of pixel change between consecutive images. Optical flow analysis can also estimate motion and relative velocity of an object through comparing consecutive frames.

An early study conducted by Bloemen et al. used activity and occupation levels to explore pigs’ responses to their microenvironment [48]. Similarly, Guo et al. applied threshold segmentation methods to detect pigs from their background in targeted areas of the pen, with potential application of predicting resource use [49,50]. Pixel analysis can also be utilized to automate detection of sick pigs [51] and has been tested on groups of pigs administered apomorphine to elicit a predictable change in locomotion levels [52]. An additional study processed subsequent frames to estimate an activity index, which was compared to human observations and found high agreement for pig activity (static activity, locomotion, and locomotion plus activity) [53].

Costa et al. described the validation of the eYeNamic system in pigs, which generates activity and occupation indices, and compared it with conventional observations by a human observer using an ethogram [54,55]. Good correspondence was found between this ‘gold standard’ and the software results, and environmental conditions including temperature, humidity, air speed and ventilation rate, were able to be related to various pig behaviours relating to thermoregulation, for example huddling (reduced occupation index) and lying spread out (increased occupation index). Kashiha et al. developed an Image Activity Status method as an alternative to eYeNamic, which performed more accurately in tests, proving to be more robust against variation in body shape; however, it still had difficulty under high stocking density situations [56]. Another potential application for image analysis of activity is the detection of the circadian rhythm. Chung et al. collected activity data at the group level in a commercial farm pen with 22–24 pigs per pen [57]. Circadian rhythm was calculated by assessing the repeatability of the 24-hour activity data across days while adjusting for pigs’ growth curve. Additionally, one study simply used webcam zone trigger software (alerted when an object entered a predefined region of interest) to track the location of an individual within a pen [58].

A more advanced monitoring method of movement involves optical flow to assess pixel changes from consecutive frames of video, but with respect to an identified object, returning more detailed information such as direction or velocity of movement. Gronskyte et al. applied optical flow analysis to assess pig welfare in the slaughterhouse by detecting abnormal (tripping or stepping on one another) versus normal movement [23,59]. Optical flow vectors from a pig were summarized into a modified angular histogram, which then allowed the application of a support vector machine (SVM) to identify movements of interest. In another attempt to detect significant changes in pigs’ motion, Fernández-Carrión et al. used an SVM on optical flow vectors from images of pigs infected with the African Swine Fever virus [60]. This method could serve as an early warning system, as changes in activity were recognized before the presence of clinical signs.

Using 3D cameras, as described above, Kulikov et al. tracked minipigs in an open field test using EthoStudio software (http://ethostudio.com/new/en/about/) [61] whereas Matthews et al. designed a system to segment and track pigs [62]. Tracking was achieved by first reducing each pig to the centre point of its detected space (centroid) and then minimising the movement of these over subsequent frames using the Hungarian algorithm [63]. Assuming that the speed of movement of pigs is not too great, a method to minimise the distance moved by pig centroids between subsequent frames is a simple but effective way to track individual pigs. The tracking was then able to produce metrics of speed and distance of movement (on the flat XY plane) and clustering (spatial entropy). The system also determined pig posture as stand or non-stand (sit, lie), which in combination with spatial location, enabled some metrics of resource use (e.g., standing at the feeder could be used to imply feeding). However, standing at the drinker could not be validated against ‘ground truth’ human video watching. An additional study employed depth video to track pigs, detect standing and lying, as well as estimating size and weight, which could serve as metrics to improve tracking ability [64].

#### Gait score estimation and posture detection

Lameness in pigs is a significant welfare concern. To develop a vision system capable of evaluating pig locomotion, Kongsro used object extraction which then generated a map image using Matlab software [65]. They then characterized structural soundness through multivariate image analysis. Alternatively, Weixing & Jin utilized image sequences captured from a side-view camera to model gait information based upon movement of body points and joint angles [66]. Motion analysis was then performed after extracting these key features in order to classify abnormal gait.

Depth information provided by 3D cameras has also been used for automatic detection of pig gait [67]. A marker was attached to each pig’s neck as it walked under a runway, and the trajectory of this marker was compared between a basic Kinect system, and a ‘gold standard’ six camera Vicon system (Vicon T20, Oxford, UK). Good agreement was found between the systems, but only if the marker was used, suggesting that the system would need further development for use in a commercial farm setting where pigs are unmarked. Two further studies detected pig posture through Zernike moments (image property representation with high accuracy for detailed shapes and magnitudes invariant under rotation), SVM (supervised learning models) [68], and thresholding depth images to detect standing [69]. Depth cameras have also been utilized to detect posture information in lactating, crated sows [70,71]. Depth cameras have the advantage of functioning in the absence of light and have been used to detect standing pigs at night [24,25]. Finally, the posture of body parts can be detected using depth information: D’Eath et al. used a proprietary machine vision system with 3D cameras to accurately (73.9%) detect pig tail posture, and validated this indicator as potential early warning sign of tail biting [8].

#### Behavioural detection

Through different combinations of segmentation techniques, location, and posture information, more complex pig behaviours can be detected through image analysis. One study was able to detect respiration by implementing a concave and convex recognition method [72]. Through use of the animal tracking program EthoVision, pigs’ lying proximity, orientation (parallel, antiparallel, or perpendicular), as well as nosing around the ear of another pig were detected [73]. In a series of papers, Nasirahmadi et al. developed a machine vision approach to record pig lying behaviour [74]. After segmentation, moving pigs were eliminated using previously discussed methods. The clustering of the remaining lying pigs was defined as close, normal, or far using Delaunay triangulation. This, along with pig location within the pen was correlated with changes in pen temperature. Nasirahmadi et al. built on this approach by using a neural network with a back propagation algorithm to find the optimal combination of different metrics from the Delaunay triangulation to best describe the different lying patterns of pigs to identify cool, ideal or warm temperatures [15]. Nasirahmadi et al. strategically altered pen activity through the provision of feed to test the performance of their algorithm [75]. In a different application of their approach, to detect mounting behaviour, Nasirahmadi et al. repeated their approach to fitting ellipses to monochrome pig outlines [76]. Greyscale thresholding failed to separate mounted pigs, as the model tried to fit an ellipse to a mounted pair as though it were a single pig. However, the resulting ellipse is then either unexpectedly long (mounting from rear), or too wide (mounting from side- T shaped outline), allowing mounted pairs to be identified. Kashiha et al. employed an innovative approach to estimate water use of fattening pigs in real-time [77]. Pigs were segmented, then body contour was analysed to identify the pig’s head. A drinking event was detected when the pig stood still for a period of at least two seconds with its head at the drinker.

Pig aggression, another behaviour critical to animal welfare and production efficiency, has been successfully detected through machine vision techniques. Oczak et al. used the output of a relatively simple pixel based ‘activity index’ as the basis for a system to detect activity indicative of aggressive behaviour (fighting) in pigs [78]. Features such as the maximum, minimum and average activity over successive seven second periods were used as inputs into a multilayer feed forward neural network. This was trained with 24 hours of video of unfamiliar pigs mixed into a new group and was able to learn the characteristic activity patterns that indicate aggression. It was over 90% accurate at identifying aggression in a different group; however, the system was not tested against other high activity events such as locomotor play by young pigs in a new pen. From the same group, Viazzi et al. weaned piglets into groups of 12 pigs per pen and recorded the interactions over 60 hours to detect aggression in pigs [79]. Aggressive interactions were labelled manually and related to the Motion History Image from which the mean intensity and the occupation index were used. Linear Discriminant Analysis was then used to classify aggressive interactions. The algorithm resulted in 89% accuracy when validated against the manually labelled data. The work on aggression was continued by Chen et al., using a vision-based method to separate fighting pigs from the other individuals in the pen [80]. Chen and co-authors studied the acceleration between adjacent frames, as aggression typically consists of rapid movements. The algorithm could reliably detect high velocity aggression (97% accuracy) and medium aggression (95.8%). There is a risk, however, that low velocity aggression is not detected accurately and that high velocity non-aggressive behaviours, such as play, are misclassified as aggression. In a contrasting approach, Lee et al. segmented standing pigs and tracked them using Euclidian distance between subsequent frames under some threshold [22], and an index algorithm developed by Zuo et al. [81]. Various movement metrics (features) were then generated (minimum, maximum, mean and standard deviation of velocity, and distance between the pigs). They then used a machine learning approach, training support vector machines with instances of different types of aggressive behaviour manually labelled by a human observer. There were two support vector machines used in sequence, one to identify aggression from non-aggression (which was 95.7% accurate in this study) and a second to identify sub-types of aggression such as head knocking (88.9%) and chasing (91.5%).

#### Individual tracking and identification

An early attempt to segment pigs and track them over multiple frames using their centre of gravity was reported by Perner [19]. This system also included components to deal with partial occlusion of pigs by pen bars. Another early attempt was conducted with limited success by Zelek & Bullock through use of blob tracking, or identifying regions in an image with significantly different visual properties (i.e., white pigs on a dark background) [82]. Ahrendt et al. developed a system to find and track individual pigs in a group of three [83]. After a human user had identified the initial start point of each pig, their location was updated frame by frame using five channels- RGB colour, and x, y co-ordinate, identifying a blob of similar pixels on the back of each pig. The inclusion of a continually-updating colour model was intended to avoid some of the problems of tracking using colour cameras such as variable light intensity, shadow and light from windows. Tracking of three pigs for eight minutes was achieved, although if pigs mounted, jumped or spun rapidly, tracking could be lost. Kashiha et al. used monochrome video to record images of 10 pigs in each of four pens that had been marked with blue paint sprayed on their backs in an individually distinctive way (lines, crosses, rectangles or blobs of paint in specific locations) [84]. Each pattern always included a triangle sprayed on the neck. Marked pigs were then segmented and an ellipse was fitted to the outline of each pig. The location (relative to the neck triangle) and shape of the paint markings was then binarised and extracted, and Fourier transforms performed to produce Fourier descriptions. The system was able to identify individual pigs with 88% accuracy, failing to assign an ID 11% of the time, and misidentifying 1%. Errors resulted from pig body posture and paint fading. Individual identification was then used in combination with spatial co-ordinates to generate data for each pig’s location in four quadrants of the pen. Similarly, individual recognition of marked piglets in the farrowing pen by video was achieved by distinguishing them based on spray marked colours [85]. Nine piglets per litter had a different colour mark by which the programme could detect the location (X, Y coordinate) of the piglets after initial manual introduction of the colours to the programme. The programme could then detect 72.5% of the piglets, of which 89.1% were correctly identified.

In a more ambitious paper, Huang et al. attempted to individually identify unmarked pigs based on the texture of the hair on their backs [86]. Seven pigs were used, which appear from photos in the paper to be quite different in colour and texture. Pigs were segmented and binarised using the method of Guo et al. [50]. The authors combined two methods of characterising texture—Gabor wavelet at a range of scales and local binary pattern for small details. Using machine learning, a structured vector machine was then trained to recognise the distinctive features of each individual pig. They were able to achieve recognition accuracy of 92%. Zhu et al. implemented machine vision to recognize drinking behaviour [87] of individual pigs within a drinking zone by colour moment features and the extraction of geometric features such as area and object contour perimeters. This method of image processing allowed for individual pig recognition without the need for individual marking of animals. Drinking behaviour was confirmed by measuring contact between the pig and the drinker nipple and time spent in contact. This top view monitoring system used a colour camera operating in real time and correctly recognized drinking events for individual pigs 90.7% of the time. An additional study that found success in tracking individual, unmarked animals in a group was conducted by Yu et al. [88]. This group similarly created an algorithm that used colour, texture, and edge features to differentiate among pigs using video from top down colour cameras. A Kuhn-Munkres algorithm was employed to generate movement and trajectory information. This system was able to track individuals with an accuracy of 92.3%. One of the benefits of this method is its robustness when pigs are in crowded situations.

### The use of automated behaviour recording in poultry

The use of image-based analysis techniques in poultry has focused primarily on detecting and tracking birds’ positions and calculating the amount of activity and their distribution pattern. The typical aim of these studies is to provide automated measures of birds’ behaviour and to link these with their health and welfare status.

#### Laying hens

The application of image-based techniques in lying hens first focused on detecting hens’ locations in the early 2000’s, and in recent years, studies have shifted towards tracking of individual hens. The final aim of both techniques is to extract features that allow specific behaviours to be characterised.

#### Location

In the literature search, we identified three studies focusing on the location of caged hens. In all of them, image pre-processing for image segmentation was performed, using techniques described in the previous sections. Leroy et al. used the reference image method [89,90], while Cronin et al. and Kashiha et al. applied Otsu’s method [91,92]. Leroy et al. aimed to develop an image-based analysis technique to recognize five different behaviour phenotypes: standing, sitting, grooming, scratching and pecking [89,90]. Using top-view monochromatic 2D images of single caged hens, an ellipse fitting technique was applied, and features such as the centroid, rotation angle and the major and minor axes were extracted. Afterwards, a dynamic linear model was used to monitor the time evolution of these features. Labelled example image sequences for the five behavioural phenotypes were used to train the model. In a validation dataset, sleeping and standing were the most accurate detected behaviours, with 96 and 90% accuracy, respectively. Difficulty in tracking hens’ heads lead to a lower accuracy (ranging between 70–96%) for pecking.

Cronin et al. developed an image-based technique to count hens’ legs in the cages and to detect foreign objects in the belt to collect eggs [91]. In order to do that, a camera, together with an infra-red light, were attached to the automatic feeder system that moves in front of the cages and also placed on top of the egg belt. 79% of hen legs and 95% of foreign objects in the egg belt were counted correctly. The IR light reflected well from chicken legs but also from reflective surfaces in the environment. In combination with the movements of the hen legs between images, this affected the accuracy of results. The image processing software was not fully described, but the authors claim that it should be possible to relate the output to other monitored variables to define activities and link them to hen health and welfare. Finally, Kashiha et al. performed an environmental preference test for hens by applying different levels of ammonia in a system built by four connected cages [92]. Top-view images from four cameras, each one on top of each compartment, were recorded. After image segmentation, the image-based method for ellipse fitting developed in Zhang et al. was used [93]. Once the ellipse was fitted, features such as centroid, orientation and major and minor axes are estimated. A negative trend between occupation level, calculated by the image system, and ammonia level in the cage was found. The image-based system was able to track a hen in different chambers with 95.9% of success rate.

#### Tracking

As in the previous section, three studies were found regarding tracking of hens using image analysis. Two of them aimed to track individual hens in small groups, and the remaining study attempted to estimate activity levels in small groups of hens.

Zakarmi et al. developed a system based on 3D images to identify and track individual hens living in small groups (five to 10 individuals) in an experimental setting [94]. The aim was to monitor behaviours such as locomotion, perching, feeding, nesting, and drinking. Images were segmented using the Watershed algorithm and morphological filtering, then the objects of interest were defined and tracked. As backup, an RFID system was used to track individual hens. The image-based processing technique sometimes lost identification when sudden movements occurred or hens were together in the nest and one suddenly leaves. In such situations, the RFID information was used to re-identify the lost hen. Thus, the fusion of these two approaches enabled both tracking of a hen and characterisation of behaviours by monitoring trajectories and time budgets. In a more recent study, Wang et al. developed an algorithm based on a Hybrid Support Vector Machine (HSVM) for automatic tracking of individual laying hens in a layer group raised in a floor system by using an experimental platform [95]. The image-based system consisted of three steps: initialization, tracking and updating. The initialization was done manually by fitting a hen’s contour in the initial image. Then, the tracking was performed using three HSVM models. A binary HSVM detected the tracking object; a regression HSVM located the target more accurately and a one-class HSVM distinguished between individual targets via an appearance model, considering a non-rigid body movement for the hen. Experimental results demonstrated that the HSVM tracker was robust, and it outperformed state-of-the art tracking algorithms such as the Frag (fragment-based tracking method), the TLD (Tracking-Learning-Detection), the PLS (object tracking via partial least squares analysis), the MeanShift, and the Particle Filter algorithms.

In summary, current image-based analysis techniques developed for lying hens have focused on the identification and tracking of individuals. These systems perform well in terms of individual hen identification, but successful long-term tracking remains challenging. Occlusion situations, together with the erratic behaviour and rapid movements of birds, make it extremely difficult to develop robust tracking systems.

#### Broilers

Image-based analysis techniques developed for broilers have focused mainly on two aspects, tracking of individuals and monitoring of activity and distribution patterns in broiler flocks. The ultimate purpose of these studies has been to automatically evaluate broiler behaviour and establish links with health and welfare.

#### Tracking

Studies focusing on tracking of individual birds in a flock have mostly been performed on small groups (< 15 birds). The first attempt was the study by Sergeant et al. [96] who developed an algorithm to be applied to 2D images. This image-processing technique used a reference image to perform background subtraction and morphological filtering. Afterwards, using the curvatures of the contour points, the centroids and regions of interest were determined to identify the different individual birds present in the image. Finally, a correspondence between these centroids was assigned over successive video images to track each individual bird’s movement. This algorithm was tested with 13 birds and proved to be accurate for centroid estimation, with only 5% of centroids showing an error higher than five pixels. However, it was not able to deal with occlusion events.

Fujii et al. developed a poultry tracking system to analyse the behaviour of chickens infected with avian influenza [97]. The algorithm was also developed on a small flock (10 birds), and similar to the previous study, a background/foreground separation was performed followed by segmentation by ellipse fitting. However, to tracking the birds, particle filtering was applied to efficiently separate chickens from each other. The particle filter was based on a model to estimate the likelihood of a certain target. The active ray and contour models used performed well when there was close contact between chickens; however, this strategy was not optimal when dealing with occlusion events. On the other hand, the particle filtering based on colour and contour features, together with information from previous trajectories, allowed discrimination between individual birds after occlusion events. However, this combined system was able to recognize and track poultry for only a limited amount of time (around 30 seconds) before errors began to accumulate. Broiler behaviour has a higher degree of randomness than, for instance, human behaviour. Thus, the development of algorithms to predict the location of broilers after overlapping or sudden changes in direction becomes harder. Therefore, the problem of tracking individual birds remains unsolved and upscaling these approaches to larger size flocks is challenging.

#### Activity and distribution estimation

There have been several studies, based on different image-based analysis techniques, which calculate activity and distribution patterns to establish a link between broiler behaviours and health and welfare. These techniques have the advantage of working at flock level without the need for individual tracking.

For example, Bloemen et al. developed an image-based technique to calculate activity and occupation levels by broilers [48]. In this work, occupation was defined as the percentage of pixels in each individual image that are above a certain defined threshold to determine whether there is an object captured by that pixel or not. Activity was defined as the amount of pixels that have changed from background to object or vice versa by comparing two consecutive images. This approach was used to evaluate the response of broilers, pigs and water fleas to changes in the environment, and was able to define accurately the occupation and activity of these species as defined above.

In the work of Kristensen & Cornou, a similar technique was employed to estimate broiler activity [98]. Their aim was to detect deviations in the activity level of groups of six broilers in experimental pens from 2D top-view images, and to link these deviations with problems affecting the broilers. The image processing technique was refined in this work; each image was divided in blocks and activity measurements were calculated for each block. Then, a multi-process dynamic linear regression was applied to remove outliers, and another dynamic linear process was used to estimate normal activity levels. Afterwards, deviations were determined using a V-shape mask approach. In these experimental conditions, the technique was able to define the daily activity patterns and to detect deviations from them. Kashiha et al. used this image-based technique to estimate the expected distribution of broilers in a commercial house [99]. An algorithm was developed to detect deviations from this expected distribution index and raise an alert for the farmer. Then, these alerts were linked to management problems in the house, such as, power failures and block feeder or water lines. This early warning system showed an accuracy of 95%. This was developed further by Peña Fernández et al. who applied the same early warning system to detect deviations in broilers activity and occupation patterns [100]. This technique was applied in a commercial setting to estimate the percentage of time broilers have spent in an alert situation throughout the growth period. The percentage of time spent in alert situation by broilers was correlated with welfare issues, such as footpad lesions and hock burns. Moreover, applying this image-based technique splitting the image in specific areas in the broiler house it was possible to provide an indication to the farmer as to which areas were affected by the event detected. Moreover, this splitting can be performed in such a way that specific behaviours, such as feeding, drinking or resting are expected to be performed more often in certain parts of the building. Combining this image-based technique with a transfer function model allows monitoring both the location of the event and its impact on a specific behaviour.

Using this image technique to calculate activity, several studies have been performed in order to determine if monitoring broiler activity is a potential way for assessing gait score level at commercial farms [101–103]. In Aydin et al., the aim was to investigate the activity levels of broiler chickens in relation to their gait scores under laboratory conditions [101], and gaits were scored on a 0–5 scale, with 0 indicating no signs of lameness and 5 indicating the most severe lameness [104]. They found that broilers with gait score three showed significantly more activity than birds with other gait scores, possibly due to their need for more feed. Broilers scored as four or five for gait showed significantly lower activity. Lighting, camera characteristics, background and test subject’s traits, all influence the ability of the system to recognize the broiler and record its movement accurately. Therefore, in Aydin et al. the previous analysis was refined by using spatial information besides activity levels [102]. Activity was calculated similarly, but colour image features are used to define spatial use by broilers. Thus, in each image a colour filter was applied to enhance the red colour component. Moreover, a threshold was applied to define the broiler position and estimate the centre of mass. Similar results as in the previous study were obtained, but also, broilers scored as zero and three for gait seemed to explore more of the building. Aydin developed a new method to assess the lameness of broilers using 3D images [103]. Depth image information was used to define a chicken entering or leaving the recording area. Then, applying a lower and upper threshold to the depth matrix, the broiler’s body was segmented. Afterwards, the contour and orientation of the broiler was estimated. Besides, using the depth information, the back posture of the chicken along the orientation axis was defined. Then, the highest point from this back posture was used for further analysis. This image-processing technique allows detecting the number of lying events (NOL) based on the information of the distance between animal and the 3D camera. In addition, latency to lie down (LTL) of broilers was estimated. Comparing these results with visually assessed manual labelling data (reference method), 93% of NOL were correctly classified from data of 250 broiler chickens. Furthermore, the results showed a significant positive correlation between NOL and gait score (*r* = 0.934) and a significant negative correlation between LTL and gait score level of broiler chickens (*r* = -0.949). Therefore, it seems that this 3D machine vision monitoring method can be used as a tool for assessing lameness of broiler chickens.

Finally, the work of Youssef et al. investigated the effect of different temperature and ventilation conditions on behaviour [105]. A small, ventilated test chamber, populated with nine seven-day old chickens, was used. A digital CCD camera was mounted on the top of the chamber to capture the birds' positions and motion. Chickens occupied zones with lower air velocities (< 0.20 m s^-1^) under cooler conditions (<29°C), preferring zones with higher air velocities when they were undergoing heat stress (>39°C). When the ambient temperature inside the test chamber deviates from the comfort zone, broilers start to move (sudden increase in the global activity index) from the zones of poor comfort conditions to others of more suitable conditions. A first-order dynamic transfer function model is suitable (*R*^*2*^ = 0.89 and *YIC* = -11) to describe this dynamic response. Moreover, changes in the estimated model parameters in relation to the changing ambient air temperature were able to reflect the thermal status (in relation to the bird’s thermoneutral zone), as well as the behaviour of the broilers.

In summary, image-based analysis techniques which depend on the number of pixels estimated to be birds, and changes in those pixels allows estimates of activity and occupation levels in broiler houses, both in experimental and commercial setups, and these have been validated with respect to specific behaviours and aspects of welfare status. However, these basic image-based techniques become limited once birds are reaching around one kilogram of weight under commercial stocking densities. As broilers become bigger, they begin to occupy most of the area covered by the camera, making activity and occupation indices less reliable. There can be a lot of activity in the flock, as there are only a few free pixels left in the image, then the activity index will be much lower than it is in reality. Due to this limitation, other techniques to estimate broiler activity and occupation have been proposed.

Dawkins et al. used a different image-based technique known as optical flow [106]. Optical flow is based in detecting the rate of change of brightness in each area of an image frame, both temporally and spatially. This allows the calculation of local velocity vectors to define this optical flow. Then, optical flow statistics, such as mean, variance, skewness and kurtosis can be used as characterizing features. In this work, this technique was tested using images from a CCTV system inside the broiler house and on-farm outcomes related to broilers welfare were obtained. For example, negative correlations between gait score and the mean and variance of optical flow were found, while positive correlations were found with skewness and kurtosis. In further work, Dawkins et al., went on to show that skew and kurtosis optical flow statistics could discriminate between the small differences in movement of broilers if they were lame or exhibiting hock burns [107]. Colles et al. used 2D colour images of broiler flocks and reported that alterations in optical flow mean and kurtosis statistical daily values could predict when a flock would go on to have a later diagnosis of Campylobacter [108]. The authors speculated that changes in behaviour due to poorer health and welfare for reasons other than Campylobacter might also be detected using optical flow. Thus, optical flow seems to be a robust, image-based technique that can be used to estimate flow movement dynamics that can be related to health and welfare issues affecting a flock. However, this technique requires high light levels to perform optimally, and it is also affected by the size of the birds and their distance to the camera lens. So far, this technique has been applied to broilers of a specific age, and further studies are needed to generalize it to other birds, sizes and stocking densities.

#### Posture characterization

Characterising the posture of broiler chickens may allow specific behaviours to be identified. Pereira et al. investigated the use of behavioural parameters to assess the welfare status of commercial broiler breeders [109]. Using an ethogram, a trained observer viewed video images and found that two to five frames were required to identify defined behaviours, such as wing spreading, bristling, drinking, scratching, resting, stretching and preening. A library of these image sequence samples was collected with examples of birds expressing each behaviour of interest. The red, green, blue (RGB) encoded image was first translated to a hue, saturation, intensity (HSI) encoded image to enhance colour contrast. Then a segmentation process was performed, followed by feature extraction of different behavioural movements and postures. Finally, a behavioural classification tree was developed using these image features, by means of the software tool Weka (model J48), and validated. This technique was then applied to differentiate body shapes from a sequence of frames as the birds expressed their behaviours. An accuracy of 70.8% in the cross-validation dataset was achieved. Lighting conditions affected the performance of the image-based technique developed. Individual tracking to use information of a bird’s trajectory is expected to improve the algorithm’s performance. Five years later, the work of Zhuang et al. aimed to developing a real-time monitoring based on posture changes in sick broilers to detect disease outbreaks [110]. Bird flu virus was inoculated intra-nasally into healthy broilers placed in isolator cages. Colour side-/angle-view video images were collected. An image-based technique was used to segment broilers from the background. Then, the outline and skeleton information of the broilers was extracted. Finally, the postures of the broilers were analysed by machine learning algorithms, and the diseased broilers vs healthy controls were predicted. Using some of the features proposed in this research, accuracy rates of 84.2%, 60.5% and 91.5% were obtained, but using all the features together with a Support Vector Machine (SVM) model an accuracy rate of 99.5% was achieved.

To sum up, flock-level analysis of broilers using image-based techniques can be done successfully. Using either activity and occupation measurements or optical flow techniques, it is possible to characterize flock dynamics and detect deviations from normal patterns, which can be linked to management, health or welfare problems affecting a broiler flock. Therefore, it is possible to gather useful information at the group-level using image-based techniques in the absence of individual tracking, which remains challenging, in terms of both identification and long-term tracking of individuals in large flocks.

### The use of automated behaviour recording in dairy cattle

In dairy cattle, there is widespread use of technology to reduce human workload (e.g., milking robots), and behavioural monitoring using image technology has been studied since the early 2000s. Therefore, this discussion of using computer vision technology in this species has been handled a bit differently than the sections on pigs and poultry, with the review examining three applied areas: reproduction, lameness and daily management.

#### Reproduction

Much research has focussed on the economically important questions of heat detection and calving in dairy cattle, to identify the optimal time for artificial insemination and to identify difficult calvings that might require human assistance, respectively. Cangar et al. monitored pre-calving dairy cows and successfully used image analysis to quantify the changes in lying and standing that indicate the onset of calving [111]. Images were recorded from the top- and side-view using 2D cameras during 6 hours within the 24-hour period prior to calving. Side-images were labelled by an ethologist and used as the gold standard to train the algorithm. Applying an algorithm, which fit a 2D model of the dairy cow body’s configuration using the top-view images, first body features were extracted then motion features were extracted over consecutive images. This work was extended to determine the need of human intervention at calving [112]. Van Hertem et al. attempted to quantify activity and calving time [113]. In this study, five different segmentation algorithms were used to extract cows’ body contours from the background of 2D side-view images when cows were walking along a corridor (algorithms listed in Table 1). It was concluded that none were suitable for reliably extracting the cow’s body contour from images with the dynamic background of the corridor. However, when cows walked in front of a solid wall, the algorithms’ ability to extract body contours significantly improved.

A system for oestrus detection using image analysis was developed by Tsai et al. [114] from 2D top-view images recorded in a roofed cow shed. Image processing was applied to define the presence of mating and oestrus events by capturing occurrences of following behaviour and mounting behaviour. The algorithm firstly detected areas with high levels of motion across video frames; then foreground segmentation and partitioning of this region of interest enabled creation of a rule to detect oestrus. While the system was successful in capturing oestrus, manual verification by the farmer of approximately 2 minutes of video was required per day to remove false positives. Finally, Ahn et al. proposed detecting the optimal time of insemination by using a support vector machine (SVM) classifier with motion history image (MHI) feature information [115]. 2D images were gathered via a real-time video stream using a fisheye camera. Area information indicating the amount of movements was then extracted from MHI, instead of motion direction, which has been widely used for person action recognition. The method was partly successful, identifying cow-mounting behaviour with a detection rate of 72%.

#### Lameness

Several studies have attempted to automatically detect lameness, which is both an economic and welfare problem that is prevalent in the dairy industry. Song et al. developed an automatic system for continuous on-farm detection and prediction of lameness using machine vision. They captured the location of the cows’ hooves to automatically calculate a hoof trackway, which was compared with human assessments of locomotion scores [116]. Recorded 2D side-view videos were split into sequences of bitmap images. After background subtraction, binary image operations, calibration and hoof separation, the trackway information containing hoof location in the real world and its related time in the video was calculated. The mean correlation coefficient of all measurements was 94.8%. Poursaberi et al. used also image analysis techniques for early lameness detection [117]. In each frame, a hierarchy background/foreground exaggeration was used to segment the cow in each frame and track it in video. Then, the back posture of each cow during standing and walking was extracted automatically, and a lameness score was generated based on back curvature. Pluk et al. designed a combined method using image analysis and pressure mat data together to identify lame cows [118]. 2D side-view images were gathered while cows moved along a corridor in which the pressure mat was set on the ground. Using this combination, touch and release angles and the range of motion was calculated for each cow. These feature variables were associated with individual lameness scores with an average accuracy of 76%. Starting with a similar approach for detecting early signs of lameness, Viazzi et al. developed a different algorithm to define an individualized dairy cow body movement pattern [119]. Again, 2D colour side-view video recordings were first gathered while cows walked along a corridor, and the back posture measurement developed by Pousaberi et al. was used [117]. However, in this study an individual threshold for lameness detection was then defined for each individual cow based on her specific movement pattern, leading to detection accuracies above 85%. Viazzi et al. then compared the efficacy of their previous 2D side-view camera system against a 3D top-view camera setup for measuring back posture [120]. Improved accuracies of 90% were achieved with the 3D setup using a validation dataset, which the authors attributed to overcoming the dynamic background issues when using the side-view 2D camera system. Further, the 3D system was able to work in real-time with a fully automatic procedure for segmentation of the images. Van Hertem et al. then assessed the 3D top-view camera system on a commercial farm [121,122], and achieved accuracies of 81.5% when consecutive measurements from an individual cow were used to classify her lameness score. Hansen et al. also used 3D top-view images from the posterior parts a dairy cow’s body to develop a method for simultaneously estimating body condition and weight and to assess lameness incidence using back curvature [123].

#### Daily management and health monitoring

Other studies using image analysis have focused on monitoring behavioural anomalies in dairy cattle that could indicate ill health. For example, animals in poor health are generally less active than healthy counterparts. Souza et al. developed software to evaluate the behaviour of confined dairy cows in free stalls by processing digitally 2D angled top-view images, then compared the results with manually labelled image sequences [124]. The software worked by mapping the RGB image to define the location of the cow, and in combination with her location in the barn, assigned the most likely behaviour to occur in that location. However, the number of cows in the image and the changing lighting and background conditions hampered the performance of this approach. Porto et al. developed a machine vision system to quantify lying, feeding and standing behaviours of cows in free stall barns [125,126]. Their aim was to relate behaviour to milk production, fetal development, oestrus detection and lameness. Four cameras were used to record overlapping 2D panoramic top-view video images from the lying area. Image segmentation and feature extraction techniques, based on the Viola-Jones algorithm, were used to determine if the cows were performing one of the three behaviours of interest. Such an approach can work well if the colour of the animal’s body animal images contrasts strongly with the background and the background is constant background. However, as dairy cattle are often patterned with a mix of white and black (or red) and the alleys and stalls of farms may be of varying colours and change with deposition of manure, conditions on commercial farms are not typically ideal. Despite this, when compared with manual labelling of the images, the method exhibited a sensitivity of 92% for lying, 86% for feeding and 87% for standing.

Our literature search revealed two descriptive papers that describe the potential to apply vision-based PLF technologies in the dairy sector. Zin et al. outlines a framework for the use of image analysis techniques [127]. Norton & Berckmans lay out a systematic approach on how to develop and implement precision livestock techniques in livestock, using image analysis to detect lameness in dairy cattle as guiding example [128]. Further, Fontana et al. and Tullo et al. used image analysis of 2D top-view images from video recordings inside dairy cows barns to validate the performance of a commercial RFID-based indoor positioning system [129,130]. The match between the labelled activity identified by image analysis and the one obtained through the positioning system varied from 92% to 97% depending on the activity. Location and activity data from this system could be extended to identify health, welfare and production status of the monitored cows, although this was not done in this study.

## Discussion

### Diversity of algorithms used

Table 1 provides a summary of the diverse array of algorithms used for image processing and behavioural association. The segmentation and feature extraction sections present algorithms related to image processing, while the section on behavioural definition groups algorithms related to approaches taken to classify or relate image variables to biologically meaningful categories. In most of the papers included in this review, segmentation algorithms refer specifically to techniques used to separate and define the object/s of interest from the rest of elements in the image, usually described as background. If the main purpose of an algorithm is to further characterize those elements, then those algorithms have been allocated in the feature extraction section in the table. However, there are many algorithms that are useful for both segmentation and feature extraction tasks, so the distinction between categories is not clear. These algorithms both segment the objects from the background and generate some information about object features as a by-product of this process. Those features can then later be used in the behavioural classification process.

It is evident from Table 1 that there has been considerable variety in the choice of initial segmentation algorithms used to separate animals from the background. The main reasons for development of so many segmentation algorithms relate to differences in background complexity and whether the background is static or dynamic. However, once the background has been removed, the choice of different morphological filters applied for feature extraction is more limited and homogeneous across studies. Afterwards, depending on the animal species studied and the segmentation aim, there are also several techniques to refine the final segmentation. They are based on previous knowledge about the shape of the intended object, colour differences and more. Even though there are a large variety of segmentation algorithms, clusters of methods based on the same working principle (but making use of it in slightly different way) can be defined.

There may be several reasons for the diversity of approaches used to automatically detect behavioural information about animals. On one hand, many studies start from scratch when addressing a particular problem, instead of building on what was already known from previous studies. In some cases this may have come about because similar studies were performed and published within a short time of one another. Alternatively, researchers may have been unable to freely access previously published work, or they may not have been able to locate relevant studies despite searching online databases. Inability to find relevant articles could be due to differences between the terminology used to search and that used in articles published in journals of different scientific or engineering fields. Further, despite increased ability to locate content online even with via searches using standard web browsers, some content may simply not be found without knowing where to look.

Another reason for the diversity of approaches used may be that the conditions under which the studies were performed also varied greatly, not only from species to species but also from experimental to farm conditions. The context in which recording takes places certainly has an impact on performance of an algorithm and may dictate which refinements or extensions need to be applied to existing algorithms.

Regarding techniques applied to perform feature extraction and establish behavioural links, it is difficult (and perhaps not even desirable) to achieve a uniform approach. The selection of one technique over another depends on the reason for which features are being extracted and the behaviours of particular interest. The animal species under consideration can also affect the selection of the methods used to detect its behaviour. For example, slower moving dairy cattle, with their distinctive black and white appearance, may be more amenable to individual tracking than densely packed finisher pigs or rapidly-moving hens.

### Next steps

#### Provide explicit details

In order to make published work on using machine vision for automated detection of behaviour more useful for those wishing to expand up on it or to apply it in practice, the reporting standards for scientific publications on the topic must improve. Specifically, many papers lack detailed descriptions in the material and methods section that would be useful for other researchers to know, such as the type of camera, exact experimental conditions or details of the type and number of animals used in the experiment. Authors may be unintentionally omitting details depending on their perspective and what they consider to be important for replicating the work. For example, a computer scientist may not consider the age or breed of an animal important while an animal scientist may omit details about lighting type or level. Authors should be encouraged to describe in more explicit detail the features of the experimental setup (e.g., lighting source and intensity, background), animals (e.g., age or weight), and other pertinent conditions (e.g., presence or absence of marks used for tracking), as this lack of information is one of the key difficulties for allowing repeatability, validation or building from a solid starting point. Articles with accurate and descriptive titles and keywords will be make it easier for new researchers to locate previously published work. Detailed description of the condition in which work was conducted would allow researchers embarking on new studies to be better informed about the advantages and/or limitations of the algorithms used previously, determine if the approach followed in that study is useful for their objectives, and to adapt accordingly to suit conditions of a new study. It is also critical to explicitly present negative results. Other fields, such as medicine and insect science [131], have recognized the importance of publishing null results and readily accept publications of this nature. By being honest about difficulties encountered, pitfalls, and limitations of approaches, naïve readers may be averted from thinking the technique will work perfectly off the shelf under any conditions, as many do after reading publications which describe their findings so optimistically.

#### Communicate and collaborate

It was striking to us as we performed this review, how rarely studies referred to the work of other labs working on similar aims in the same species, and instead authors referred more to work published on general image analysis. Going forward, we recommend that researchers perform a thorough search of the literature to locate publications on previous work performed in their area of interest to avoid repetition and to learn from what has worked previously and what has not. Such searches should be performed not only in the disciplinary journals with which the researchers are familiar but also by using broader search engines or deliberately searching journals of other relevant fields related to using machine vision to detect behaviour.

We encourage collaborative research and publication among machine vision researchers (e.g., computer scientists, engineers, etc.), who can provide expertise on the algorithm choices and development, and animal scientists, who can provide expertise on the study animals and their housing, husbandry and sample sizes needed for biological validation. Increasing communication and cooperation between research groups working on behavioural detection within a species will provide a more robust perspective on the problems to be solved and on solutions that are practical so that progress can be made more quickly, and it seems there is a trend towards achieving this goal (e.g., [8,15,60]).

#### Scale up and expand

Research in automated behaviour detection using computer vision has mainly been carried out in experimental situations and with a limited number of animals. However, if the goal is to scale these techniques up to work on commercial farms, it will be important to keep in mind the on-farm conditions that will influence the performance of methods developed and tested at experimental level. Increasing the size of animal groups from several animals to several hundred or thousand animals will introduce additional challenges for automated detection techniques (e.g. [107]). For instance, individual tracking techniques suffer when occlusion events happen, such as when one animal mounts or rests on another (e.g. [56]). More animals in a group will lead to an increased number of occlusion events, greatly affecting the performance of individual tracking algorithms. Furthermore, larger spaces on real farms can add further challenges as regards image resolution, ‘fish-eye’ lens distortion or combining images from multiple cameras (e.g. [115]). Thus, effort should focus on defining which aspects of the developed methods are affected by scaling up from experimental to commercial level and how to improve them (e.g., incorporating RFID sensors in complement with image analysis), rather than refining details at experimental level that may already be robust enough for commercial settings.

### Knowledge gaps

As seen in this review, machine vision approaches for monitoring animals and their behaviour are developing at a rapid pace. This is not only the case for farm animals, but also domestic animals (pets and working dogs) and wildlife (reviewed by Jukan et al. [132]). Some of the difficulties faced in the early stages of automatic detection of behaviour using machine vision have been solved by the availability of better quality and more advanced cameras, such as 3D and high speed. There are, however, still plenty of knowledge gaps to address. One of the current key knowledge gaps is how to track individual animals in a pen and record their behaviour while continuously recognizing the individual, especially when dealing with unmarked animals without wearable sensors. In dairy cattle, these problems can be overcome by, for example, identifying individuals based on coat patterns; however, this option is less viable for more nearly identical looking animals such as all-white laying hens, except in test settings where the manual labelling of a few animals is possible. Combining methods of automated video analysis with other on-going technological advancements may in future allow us to overcome this problem. For example, animals’ faces can be recognised when they come to the drinker or feeder space, allowing tracking of an unmarked animal for a limited amount of time thereafter [77], and facial recognition of pigs is being used in practice in China and also in Europe [133].

Another challenge is to validate the algorithms in larger groups of animals. As mentioned in the section on limitations, many of the reviewed studies had small sample sizes. It has yet to be demonstrated whether the algorithms that work well at the small scale can perform as well when the number of animals per pen increases to levels typically housed in commercial operations. Sharing of algorithm information to enable testing by multiple research groups will increase the true accuracy across contexts. However, confidentiality of such information may be a limiting factor.

### From experimental tests to on farm application

At this stage, moving from using machine vision to automatically detect the behaviour of a few animals in a test pen to recording hundreds or thousands of animals housed indoors in a commercial situation is still a hurdle to overcome for many approaches. However, other Precision Livestock Farming technologies have already made their way into commercial practice. Sensors, for example, are now common in the dairy sector (reviewed by Neethirajan [134]). Audio surveillance systems are also gradually making their way onto farms as a way of detecting disease, particularly respiratory diseases, at an early stage (e.g., pigs: [135]; [136]; poultry: [137]; cattle: [138]). In fact, an automated sound detection system (SoundTalks) can detect the onset of disease better than humans can, and therefore use of technology enables more timely and efficient treatment [139].

The ability to move automated detection of behaviour using machine vision into practice on farm for indoor-housed animals still has some practical obstacles. Even if all technical difficulties with image analysis under varied farm conditions were solved, the implementation of automated behaviour analysis would still depend partly on considerations related to the cost and maintenance of cameras. For example using machine vision to count piglets is useful on farm for monitoring mortality [38], but requires a video camera above each pen (or every few pens). In poultry, a few cameras can be used to cover a whole barn for detecting optical flow patterns, from which flock behaviour related to welfare issues can be recognised [107]. However, in all cases cameras in barns need maintenance—at the very least to regularly remove dust and insects from lenses to ensure unimpaired vision. However, the benefit of being able to automatically detect behaviour can outweigh the costs and effort of camera installation and maintenance; for example as when damaging behaviours such as cannibalism in laying hens and tail biting in pigs that result in major production losses and costs could be deterred by early detection. The development of automated detection of low hanging tails in pigs [8], a precursor for tail biting outbreaks, is therefore a promising avenue for having cameras routinely recording on pig farms. In the future, as machine vision technology and cameras get cheaper, and as systems are integrated to offer multiple benefits in one package (such as accurately counting, identifying, and weighing/condition-scoring animals while detecting a range of production, health and welfare issues including deviations from normal posture, gait, location or behaviour), these combined benefits will begin to outweigh their cost, making such systems more viable. Other than the cost / benefit issue, there are other factors which affect the uptake of PLF by farmers. The ‘mindset’ of farmers as innovators vs. traditionalists, the scale of their farming enterprise, and the perceived amount of time taken to engage with decision support systems [140]. As such we agree with Van Hertem and others [141] that it is crucial to engage with the end-users of PLF technologies, including farmers, vets and advisors, to develop user-friendly and informative decision support systems, with real-time data visualisation, supplied to the devices that they find convenient to use (smartphone, tablet or PC). There is also a need for further social science research into understanding the possible behaviour change barriers to PLF uptake by these end-users [142].

## Conclusion

In conclusion, a range of algorithms have been used to approach problems related to using machine vision to automatically track animal behaviour. The diversity of approaches reflects, in part, the diversity of research aims and number of species being studied. However, it is also symptomatic of a lack of communication among research teams or disciplines working in the relatively young space of precision livestock farming. Nonetheless, important advances have been made in using machine vision approaches to detect behaviours of interest in indoor-housed poultry, pigs and dairy cattle. Machine vision research in the poultry sector has to date typically focussed on the question of how to monitor large animal group sizes. As shown in the review, these applications quantify the apparent motion and distribution of the animal group from the brightness pattern within a 2D video. This inevitably results in lost information on motion of individual animals and future effort should aim at achieving multiple object (bird) detection and monitoring in such machine vision applications. For monitoring of pigs, typically done in much smaller group sizes than chickens, machine vision research has now achieved more accurate link between group-level image features and ethograms of important welfare related behaviours. Further work is needed to link this automatic detection with individual tracking and automatic identification of the perpetrators and victims of poor behaviour. Finally, for dairy cattle the focus has been mostly on monitoring animal motion on an individual level. In this case significant progress has been made in identifying and extracting informative image features linked with gait. In this field, research algorithms that combine gait monitoring with individual identification and social behaviour of cattle should be the focus of future research.

There are three important next steps that could benefit the progress of the research in this field. Firstly, researchers are encouraged to search thoroughly to find previous work done in relation to their question of interest to avoid unintentional repetition, and instead use knowledge of what has previously been done to build on the successes and failures of others. The increasing number of recent reviews on aspects of PLF [143–146] can play a major contribution here. Second, improving the reporting standards in scientific publications related to using machine vision for automated detection of behaviour will make articles more useful and informative so other researchers can and will use them as they progress in their own work. Finally, it will be beneficial to potentiate communication and collaboration between research groups with similar interests, but potentially different perspectives and expertise, to speed our ability to problem solve. Taking these steps will allow us to focus on addressing critical knowledge gaps, such as identifying and tracking nearly identical looking unmarked animals in large groups, and in moving machine vision applications more rapidly and efficiently from experimental to practical settings.

## Supporting information

S1 Table Legend used for retrieving data from selected publications. (PDF)

S2 Table Data file. (XLSX)

S1 PRISMA checklist PRISMA 2009 checklist. (DOC)
