# Supplementary material for: Recording behaviour of indoor-housed farm animals automatically using machine vision technology: A systematic review
Source: PLoS One. 2019 Dec 23;14(12):e0226669. doi: 10.1371/journal.pone.0226669 (PMC6927615; doi:10.1371/journal.pone.0226669)
Supplement: S1 Table — (PDF) [file pone.0226669.s001.pdf]

| Information | Type of input | Description |
|---|---|---|
| Author Year Title | Text | Basic information about the paper |
| Objective of the study | Text | Aim(s) mentioned in the paper |
| **Methods** | | |
| Species | Drop-down list | Animal species |
| Breed | Text | Breed, if mentioned |
| Age of animals | Number | Age in days/weeks/months |
| Housing type | Drop-down list | Description of housing type, Indoor, test pen, commercial |
| Floor colour / type | Drop-down list | Floor characteristics including material and colour |
| Lighting | Text | Type and strength of light source |
| Group size tested on | Number | Number of animals per pen |
| Total sample size | Number | Total sample size analysed |
| Device type | Drop-down list | Choice of: colour camera; 3-d; infrared; colour, infrared and 3D; RFID detectors under floor grid and 3D; black and white camera; Thermal camera (exclude); Black and white camera and colour camera |
| Camera model | Text | Camera specs |
| Lens model | Text | Type of lens, if mentioned |
| Camera location | Drop-down list | Choice of: ceiling, angled view; Ceiling, top down; Wall; Tri-pod; top down and side view; unknown; Top down; Side view |
| Track within group | Yes / no | Whether the method is able to track an individual within a group of animals |
| Track unmarked | Yes / no | Whether the method is able to track an unmarked individual |
| Behaviour studied | Drop-down list | Type of behaviour studied |
| Algorithm used | Yes/no | Whether an algorithm is being applied |
| Data processing | Text | Description of the data processing method |
| **Results** | | |
| Accuracy | Number | |
| Precision | Number | |
| Specificity | Number | |
| Sensitivity | Number | |
| Reliability | Number | |

| | | |
|---|---|---|
| Generated from study | Drop-down list | The type of data or outcome that is generated |
| End result | Drop-down list | Type of end product, for example an algorithm or dataset |
| Ready to use | Drop-down list | Whether the method is ready to be used by others |
| Access to data | Drop-down list | Statement about data / algorithm availability if mentioned |
